# Integrating single-channel EEG neurofeedback into video game-based digital therapeutics for ADHD

**DOI:** 10.1186/s12984-026-01918-7

**Published:** 2026-03-05

**Authors:** Chenyu Yan, Yuhan Liu, Jiajing Zhao, Mengyi Bao, Qing Zhou, Shang Feng, Haifeng Li, Gang Pan, Lin Yao, Yueming Wang

**Affiliations:** 1 Department of Rehabilitation, Children’s Hospital, Zhejiang University School of Medicine, National Clinical Research Center for Children and Adolescents’ Health and Diseases, 3333 Binsheng Rd, Hangzhou, 310052 China; 2 Nanhu Brain-Computer Interface Institute, Hangzhou, China; 3 MOE Frontiers Science Center for Brain and Brain Machine Integration, Zhejiang University School of Medicine, Hangzhou, 310058 China; 4 Department of Neurobiology, Affiliated Mental Health Center, Hangzhou Seventh People’s Hospital, Zhejiang University School of Medicine, Hangzhou, China; 5 SDO Digital Therapeutics Innovation Center, Shanghai, China; 6 College of Computer Science and Technology, Zhejiang University, Hangzhou, China; 7 College of Biomedical Engineering and Instrument Science, Zhejiang University, Hangzhou, China; 8 State Key Laboratory of Brain-Machine Intelligence, Zhejiang University, Hangzhou, China

**Keywords:** ADHD, Digital therapeutics, Neurofeedback, EEG

## Abstract

**Background:**

Digital therapeutics have emerged as a promising non-pharmacological intervention for children with attention-deficit/hyperactivity disorder (ADHD). Personalized adaptation is key to the success of digital therapeutics. However, most existing systems depend solely on observable performance rather than real-time internal attentional state, which lead to misinterpretation or delayed adaptation.

**Methods:**

In this study, we evaluated the effects of a tablet-based attention training game with and without EEG-informed real-time neurofeedback in children with ADHD. Participants were assigned to one of two groups: a neurofeedback group (NFb) in which the game adapted in real time based on single-channel frontal EEG signals and a standard game intervention group without neurofeedback (n-NFb). Attention and cognitive control were assessed before and after a one-month intervention.

**Results:**

All children showed improvements in attention in both parent report and children’s performance in attentional tasks. The NFb group showed greater improvements in hitting accuracy (go trials) and less reductions in inhibition accuracy (no-go trials) than the n-NFb group. Both groups had significantly shorter reaction times after training. EEG analyses revealed greater improvement in attention index during training for NFb group.

**Conclusion:**

Our findings suggest that video game-based digital therapeutics with EEG-informed real-time neurofeedback can effectively enhance attention in children with ADHD. The results support the potential of using adaptive neurofeedback with portable devices to enhance intervention effects.

## Key points

- (what’s known) Game-based digital therapeutics (DTx) have shown promise in treating ADHD, but their effects are often limited with inflexible adaptation strategies.
- (what’s new) This study integrated real-time single-channel EEG neurofeedback into a tablet-based DTx system, enabling real-time adaptation with neurofeedback based on internal cognitive states.
- (what’s new) A self-developed attention index provided an operational estimate of moment-to-moment attention levels via portable EEG.
- (what’s relevant) These findings support the clinical use of low-cost neuroadaptive systems as personalized, home-based interventions for ADHD, and offer a framework for future research on real-time neural feedback in digital therapeutics.

## Introduction

Attention-deficit/hyperactivity disorder (ADHD) is one of the most prevalent neurodevelopmental disorders in childhood, affecting approximately 7% of children worldwide [27]. Characterized by persistent inattention, hyperactivity, and impulsivity, ADHD significantly impairs academic performance, social interactions, and overall quality of life [33]. While pharmacological treatments remain a cornerstone of ADHD management, concerns about side effects, limited long-term adherence, and the need for non-invasive alternatives have fueled interest in non-pharmacological interventions, including cognitive training, behavioral therapies, and emerging digital therapeutics [22, 25].

Among non-pharmacological interventions for ADHD, game-based digital therapeutics (DTx) have great potential due to their accessibility, scalability, and minimal side effects [23]. Delivered through mobile devices such as tablets or smartphones, these interventions can be implemented at home with minimal supervision and are particularly appealing for children. For example, EndeavorRx, the first FDA-approved digital therapeutic for ADHD, demonstrated superior outcomes compared to treatment as usual, including environmental and psychosocial support [16]. Clinical trials (STARS-ADHD RCT) have shown that EndeavorRx significantly improves attention in children, with even greater effects observed in adolescents and adults (Adolescent M ≈ 2.6; Adult M ≈ 6.5). Japan has introduced a localized product, SDT-001 (EndeavorRide), which has completed Phase III clinical trials and received regulatory approval [21]. In China, the MindPro1 platform demonstrated significant efficacy and good safety in a pilot trial with 52 children [14]. Recently, the Chinese NMPA approved the first domestically developed DTx product for ADHD—Attention Enhancement Training Software (Approval No. 20232210315). These advances reflect the increasing international uptake of digital therapeutics for ADHD across diverse healthcare settings.

Despite promising results, clinical trials and real-world applications have revealed substantial variability in treatment outcomes, with some children benefiting greatly and others showing only modest or no improvement [23, 32]. What accounts for this variability? One of the key limitations of current DTx systems is their reliance on external behavioral indicators—such as accuracy, response time, or task completion rates—to adapt the gameplay experience. While these metrics provide valuable feedback, they may fail to capture the moment-to-moment fluctuations in attentional engagement that characterize ADHD. Children with ADHD often exhibit rapid shifts in attention driven by fatigue, motivation, and environmental context [4], which remain invisible to traditional DTx systems. As a result, interventions may fail to adjust in real time to a child’s internal cognitive state, reducing engagement and effectiveness.

Neurofeedback (NFb)—a technique that provides real-time feedback of brain activity—offers a compelling solution to this problem. By measuring ongoing neural signals (typically via EEG) and translating them into adaptive stimuli, NF empowers children to self-regulate attention and executive control processes [8, 13]. Research has shown that frontal EEG activity, particularly in the alpha and beta frequency bands, reflects attentional states and cognitive engagement [15, 31]. Traditional NF systems, however, are often complex, clinic-bound, and require multi-channel EEG setups, limiting their scalability and accessibility for pediatric populations. Recent advances in single-channel EEG technology provide an opportunity to overcome these barriers. Portable, user-friendly, and cost-effective, single-channel EEG systems can reliably detect attention-related brain dynamics, especially in the prefrontal cortex—a region critical for attentional control [17, 28]. These systems open the door to integrating real-time neurofeedback into digital games, enabling neuroadaptive interventions that respond dynamically to each child’s attentional state, rather than relying solely on behavior.

In the current study, we investigated the efficacy of such a neuroadaptive approach by integrating real-time single-channel EEG-informed neurofeedback into a tablet-based attention training video game for children with ADHD. Participants were assigned to either a NFb group, in which gameplay was modulated by frontal EEG signals, or a control group that received the same game without neurofeedback. We assessed behavioral and cognitive outcomes before and after a one-month intervention, focusing on whether neurofeedback enhanced attentional gains beyond standard training, and whether baseline attentional capacity predicted intervention outcomes. Our goal was to explore whether adapting digital therapeutics to a child’s real-time brain state could improve effectiveness and offer a more personalized, scalable intervention for ADHD.

## Methods

### Participants

A priori power analysis was conducted using G*Power 3.1 [9] to determine the minimum sample size. Assuming a medium effect size (*f* = 0.25; [6]), an alpha level of 0.05, and a power of 0.95, the analysis indicated that at least *N* = 54 participants would be necessary for a mixed ANOVA with two groups and two measurement points. To ensure robust statistical power and accommodate potential attrition, variability in effect sizes, or subgroup analyses, we recruited a substantially larger sample.

All children were recruited through the outpatient clinic of the Department of Rehabilitation at the Children’s Hospital, Zhejiang University School of Medicine. Inclusion criteria were (1) age 6 to 12 years, (2) diagnosed with attention-deficit/hyperactivity disorder (ADHD) according to DSM-5 criteria [1], (3) having an IQ ≥ 70, verified by Raven’s Progressive Matrices test [26], (4) normal or corrected-to-normal vision and hearing. Exclusion criteria were (1) use of medications for clinical treatment of ADHD, or other drugs known to affect the central nervous system’s metabolism or functioning in recent 2 months, (2) comorbid disorders including conduct disorder, and depressive disorder but only in case of current suicide risk or active suicidality, (3) current suicide risk, or with a history of suicide attempts, or previous suicidal ideation, or self-injurious behaviors assessed by licensed clinicians, (4) physical conditions impairing gameplay (e.g., deformities of the hands or arms, use of prosthetics), (5) history or suspected history of substance abuse or dependence, (6) diagnosis of color blindness.

For 84 children recruited, 6 were children excluded for using medication during the intervention, and 4 children were excluded due to missing EEG recordings caused by tablet malfunction. Finally, a total of *N* = 74 participants were included in the analysis. Table 1 presents the demographic and clinical profiles of children of all study groups. The study was approved by the ethics committee of the Children’s Hospital, Zhejiang University School of Medicine (2022-IRB-0299-P-02).

**Table 1:** Basic information of participants in groups

*N*	NFb group	*n*-NFb group
	37	37
Age	7.8 (1.5)	7.9 (1.5)
Gender (male/female)	29/8	32/5
IQ	113.9 (17.4)	113.3 (14.4)
Comorbid ASD	6	4
ADHD medication	0	0
TOVA baseline	-3.6 (3.1)	-4.1 (2.5)

Note. Values are presented as mean (SD) and categorical variables are presented as N

### Study design

**Fig. 1:**
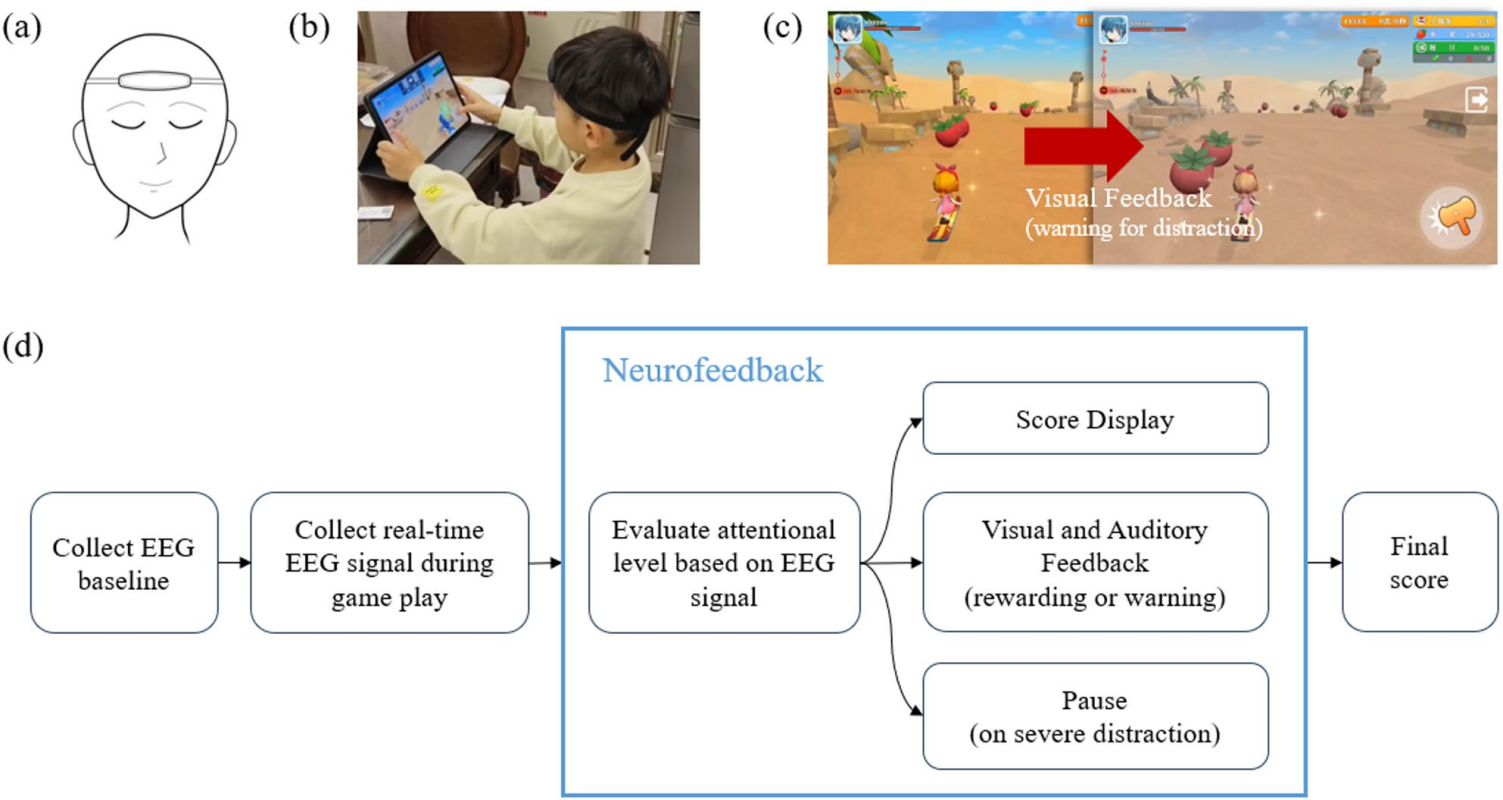
**a** The single-channel EEG device; **b** the gameplay environment; **c** illustration of visual feedback, specifically the whole display turned grey when detecting distraction; **d** overall design flow for EEG use during game play

Participants were assigned to one of two intervention groups: a neurofeedback (NFb) group and a non-neurofeedback (n-NFb) group sequentially based on recruitment order to maintain comparable group sizes. Participants in the NFb group first underwent an attention assessment at the research center, which included both task-based evaluations for the child and parent-reported measures. They were then provided with a tablet pre-installed with the training software and a head-mounted single-channel EEG device (Fig. 1a). Over the following month, participants engaged in at-home attention training using the tablet and EEG device. A 4-week digital intervention was administered, comprising 25-minute daily sessions (5 days/week). Upon completion of the training period, they returned to the research center for a post-intervention attention assessment. The procedure for the n-NFb group was almost identical to that of the NFb group. The only difference was that the intervention content on their tablets was not modulated by EEG signals. Participants in this group also completed the same attention assessments at the research center before and after the intervention period and followed the same at-home training schedule using the tablet device and EEG device.

### General game design

The tablet-based game was designed collaboratively by the Zhejiang University and Digital Medicine Intelligence Company. The game, structured as a progressive-level system, with scenarios varying across deserts, towns, villages, and volcanoes. Each level integrated three task types—egg, number, and fruit—requiring rapid target discrimination (touch/click responses) under time constraints while inhibiting responses to nontarget distractors. In the egg task, targets appeared abruptly from one side of the screen, whereas in the number and fruit tasks, stimuli moved gradually toward the player until they were reached. Level progression was contingent upon completing a predetermined number of tasks within specified accuracy thresholds. Performance data, including reaction times (RT), accuracy rates, and error types (commission/omission), were recorded automatically by the tablet system for offline analysis.

Both experimental groups wore a single-channel EEG device throughout the gameplay (Fig. 1b). In the n-NFb group, the EEG data were collected purely for recording purposes and had no effect on the game experience. In contrast, the NFb group received real-time feedback based on their brain activity (see 2.4 for details). The system calculated individualized attention metrics from the EEG signals, such as real-time attentional levels, and used these to modulate the game environment. When participants demonstrated sustained attention above their baseline levels, the game provided positive reinforcement through visual and auditory cues. Conversely, if attention levels dropped below a threshold or excessive movement was detected, the game reduced feedback intensity (e.g. the whole display turned grey) or temporarily paused gameplay. This neurofeedback mechanism was designed to encourage participants to maintain optimal focus during the game. The overall game flow was illustrated in Fig. 1d.

### Neurofeedback

In this study, an entire session of neurofeedback training consisted of two stages: (1) resting-state baseline collection; (2) neurofeedback training. Single-channel EEG device was turned on for recording and placed on the subject’s forehead prior to the experiment. During the resting-state baseline collection stage, participants were asked to sit quietly, staring at the cross located at the center of screen for 2 min. During the training stage, participants were asked to finish the designed video game tasks with neurofeedback training.

The parameters used for neurofeedback include “Attention Index” and “Movement Index”, based on the findings of a previous study [13].

Attention Index: Beta-band activity has been positively linked to attentional engagement, and has served as an effective target in broad EEG neurofeedback training [5]. While most studies used fixed beta range (~ 13–30 Hz), recent work support the use of individual beta range, given high variability in EEG spectral profiles in young population [13]. The individual beta range was defined based on individual alpha peak frequency (iAPF), a highly stable trait that has been used in personalized intervention in ADHD [2]. Thus, we used the rolling-smoothed band power of the individual beta (iBeta) band based-on iAPF as the Attention Index for neurofeedback. Specifically, the Attention Index was calculated in the following order:

(1) Calculate the “individual alpha peak frequency” (iAPF):

$$\:iAPF=\frac{\sum\:_{f=7\:Hz}^{14\:Hz}S\left(f\right)*f}{\sum\:_{f=7\:Hz}^{14\:Hz}S\left(f\right)}$$

where *f* indicates the frequency points within 7–14 Hz and *S(f)* is the power spectrum of EEG.

(2)  Calculate the division of iBeta band. The lower limit of iBeta band is 1.3 * iAPF, and the higher limit of iBeta band is 3.0 * iAPF.
(3) Calculate the rolling-smoothed PSD of iBeta band using 2-sec segments with 1-sec sliding window, with stepsize of 1 s.

Movement Index: We used the relative power of electromyogram (EMG) as the Movement Index, calculated as follows:

$$\:{Power}_{EMG}=\frac{\sum\:_{f=40\:Hz}^{125Hz}S\left(f\right)-\sum\:_{f=48\:Hz}^{52\:Hz}S\left(f\right)}{\sum\:_{f=4\:Hz}^{125Hz}S\left(f\right)-\sum\:_{f=48\:Hz}^{52\:Hz}S\left(f\right)}$$

where the *S(f)* is the power spectrum of each segment and the power of line noise (48–52 Hz) was removed from the power within each band.

Baseline values for the Attention Index and Movement Index were computed from EEG signals aggregated over the entire baseline collection stage (2-min). During the neurofeedback training stage, the Attention Index and Movement Index were calculated every second (2-sec window) and the feedback elements were presented in the training software, according to the relative ratios (comparing to baseline value) of the two Indices.

There were three types of feedback elements: (1) visual negative feedback; (2) visual and auditory positive feedback; (3) large movement feedback. The “visual negative feedback” used the visual saturation of screen color as the indicator of real-time attention. If the participant’s real-time Attention Index was lower than threshold ( 0.8 * baseline value), the saturation of screen color would be altered to the ratio of real-time to baseline. Therefore, the participants would realize their inattention condition immediately during training. The modulation of saturation only affected the background scene color, while other task-related elements were not influenced. The “visual and auditory positive feedback” used game-like rewarding element (visual and auditory effect of the avatar) to provide positive feedback when the participants sustained high Attention Index (typically set to 1.5 * baseline value) for a certain period (3 s). The “large movement feedback” used Movement Index to detect large movement of the participants. When a large movement was detected (3 * baseline value for continuously 3 s), the training process would be paused, and a prompt text would appear on the screen asking the participants to reduce their physical movements.

### Clinical attention task for children

To objectively evaluate children’s attentional changes resulting from the intervention, we applied the Test of Variables of Attention (TOVA) before and after training (within one week). TOVA is a computerized and validated neuropsychological assessment widely used for diagnosing ADHD and evaluating intervention outcomes [10, 23]. It is designed to capture core components of executive functioning, particularly sustained attention and inhibitory control. The task employs a two-phase paradigm with varying target-to-nontarget ratios, requiring participants to respond only to target stimuli while withholding responses to nontargets. A composite measure, the Attention Performance Index (API), integrates vigilance, impulsivity, and processing speed into a standardized T-score. Higher API scores indicate superior attentional functioning. The TOVA has demonstrated high sensitivity and specificity in distinguishing ADHD-related deficits from neurotypical performance, and its validity is supported by correlations with other executive function measures as well as its ability to differentiate ADHD from related conditions. TOVA administration followed standardized procedures, and outcome scoring was performed automatically by the TOVA system.

### Parental report of children attention

To complement the task-based assessment and capture changes in everyday behavioral symptoms, we used the Chinese version of Swanson, Nolan, and Pelham Rating Scale (SNAP-IV) as a parent-report measure. The SNAP-IV assessed parent-reported ADHD-related behaviors of the participants within the month preceding the study [11, 30]. This 26-item version covers the core symptom domains of ADHD—inattention and hyperactivity/impulsivity—as well as oppositional behaviors characteristic of oppositional defiant disorder. Each item was rated on a 4-point Likert scale ranging from 0 (not at all) to 3 (very much), with higher subscale scores indicating greater severity of ADHD and oppositional symptoms. Average scores above 1.5 usually indicates a high risk of ADHD.

## Result

### Children’s performance on clinical attention task

The TOVA results were analyzed using a two-way ANOVA, with treatment method (NFb and n-NFb) and test phase (pre- vs. post-intervention) as independent variables, and the TOVA API as the dependent variable. After one month of intervention, there was a significant main effect of test phase, with higher overall scores after training (*F* = 15.207, *p* < 0.001, $\:{\eta\:}_{p}^{2}$ = 0.174). In addition, there was a significant main effect of treatment method, with higher overall scores in the NFb group compared to the n-NFb group (*F* = 4.143, *p* = 0.045, $\:{\eta\:}_{p}^{2}$ = 0.054). The interaction effect between treatment method and time approached marginal statistical significance (*F* = 3.924, *p* = 0.051, $\:{\eta\:}_{p}^{2}$ = 0.052). The proportion of participants whose post-test TOVA scores exceeded their pre-test scores was 78.3% in the NFb group, and 59.5% in the n-NFb group.

Given the substantial variability in baseline TOVA performance, a baseline-adjusted ANCOVA was conducted to provide a more direct test of post-intervention group differences. In this model, post-intervention TOVA API was entered as the dependent variable, group as the fixed factor, and baseline TOVA API as a covariate. After adjusting for baseline scores, the NFb group showed significantly higher post-intervention TOVA API than the n-NFb group (*F* = 7.095, *p* = 0.010, $\:{\eta\:}_{p}^{2}$ = 0.091). Baseline TOVA API was a strong predictor of post-intervention performance (*F* = 63.442, *p* < 0.001, $\:{\eta\:}_{p}^{2}$ = 0.472).

**Fig. 2:**
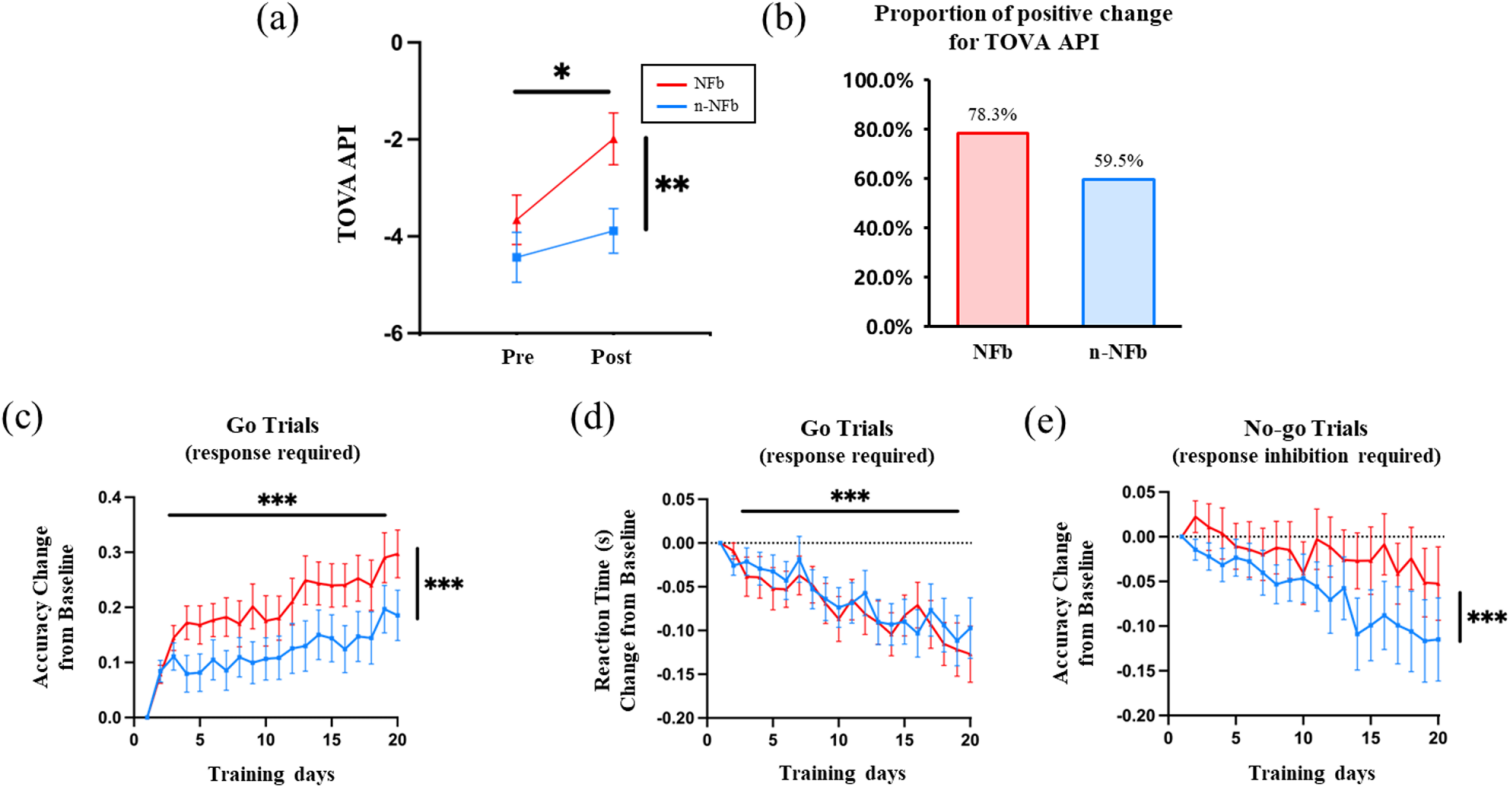
**a** Mean pretest and posttest score for TOVA Attention Performance Index for each group. **b** Proportion of positive change for TOVA API for each group. **c** Mean accuracy and **d** reaction time change from baseline for go trials (response required). **e** Mean accuracy change from baseline for no-go trials (response inhibition required). Accuracy change represents the absolute difference in accuracy relative to the first training day. Error bars represent the standard error. *p < 0.05, **p < 0.01, ***p < 0.001

To examine potential baseline effects, baseline TOVA API scores were first compared between groups and did not differ significantly between the NFb and n-NFb groups (independent t-test, *p* = 0.578). Correlation analyses showed that baseline TOVA API were negatively associated with TOVA change (*β* = −0.373, *p* < 0.001), indicating that children with lower baseline scores tended to show greater improvement overall. Notably, the mean baseline TOVA API was numerically higher in the NFb group but even showed greater improvement. Additionally, regression analysis (TOVA API change ~ baseline + group + baseline×group) revealed significant main effects of baseline and group, but no significant baseline × group interaction (*p* = 0.157)), indicating that baseline performance did not differentially predict change between groups. Accordingly, baseline differences cannot account for the greater post-intervention improvement observed in the NFb group (Fig. 2).

### Parental report of children’s attention

The SNAP-IV results were analyzed using a two-way mixed ANOVA, with treatment method (NFb and n-NFb) as the between-subjects factor, time (pre- vs. post-intervention) as the within-subjects factor, and SNAP-IV scores as the dependent variables. After one month of intervention, mean attention deficit scores significantly decreased across both groups (*F* = 4.194, *p* = 0.042, $\:{\eta\:}_{p}^{2}$ = 0.029), whereas mean hyperactivity/impulsivity scores showed no significant change (*F* = 1.974, *p* = 0·162, $\:{\eta\:}_{p}^{2}$ = 0.014). Between-group comparisons and interaction indicated no significant differences in mean attention deficit (*p*s > 0.05).

### Training performance

The performance during training on tablet game were analyzed using Two-way ANOVA with the treatment methods (NFb and n-NFb) and the training days (0–20 days) as independent variables and the change of accuracy and reaction time from baseline as the dependent variables. The results for target trials (response required) and non-target trials (response inhibition required) were analyzed sperately.

For target trials, which required rapid and accurate response, the results showed that after 20 days of intervention, the accuracy of the egg hitting task in both group significantly improved (*F* = 3.798, *p* < 0.001, $\:{\eta\:}_{p}^{2}$ = 0.047). Moreover, the accuracy of NFb group was significantly higher than that of n-NFb group (*F* = 43.520, *p* < 0.001, $\:{\eta\:}_{p}^{2}$ = 0.029). The accuracy of number hitting task was significantly improved (*F* = 5.186, *p* < 0.001, $\:{\eta\:}_{p}^{2}$ = 0.064), and the accuracy of n-NFb group was significantly higher than that of NFb group (*F* = 33.910, *p <* 0.001, $\:{\eta\:}_{p}^{2}$ = 0.022). The accuracy of the fruit hitting task was significantly improved (*F* = 10.740, *p <* 0.001, $\:{\eta\:}_{p}^{2}$ = 0.127), but there was no significant difference in accuracy between n-NFb and NFb group (*p* = 0.119). The reaction time to respond to the target in the egg hitting task was significantly shortened (*F* = 3.779, *p <* 0.001, $\:{\eta\:}_{p}^{2}$ = 0.050), but there was no significant difference in the reaction time between n-NFb and NFb group (*p* = 0.345).

For non-target trials, which required inhibition of response, the results showed that after intervention, there was no significant change in the non-target hitting accuracy of the egg hitting task between n-NFb and NFb group (*p* = 0.136), and the accuracy of NFb group was significantly higher than that of n-NFb group (*F* = 19.220, *p <* 0.001, $\:{\eta\:}_{p}^{2}$ = 0.013). The non-target accuracy of number hitting task was significantly improved (*F* = 4.710, *p <* 0.001, $\:{\eta\:}_{p}^{2}$ = 0.060), but there was no significant difference in the accuracy between n-NFb and NFb group (*p* = 0.990). The non-target accuracy of the fruit hitting task was significantly improved (*F* = 2.890, *p <* 0.001, $\:{\eta\:}_{p}^{2}$ = 0.041), and the accuracy of NFb group was significantly higher than that of n-NFb group (*F* = 7.737, *p* = 0.006, $\:{\eta\:}_{p}^{2}$ = 0.007).

### EEG analysis

**Fig. 3:**
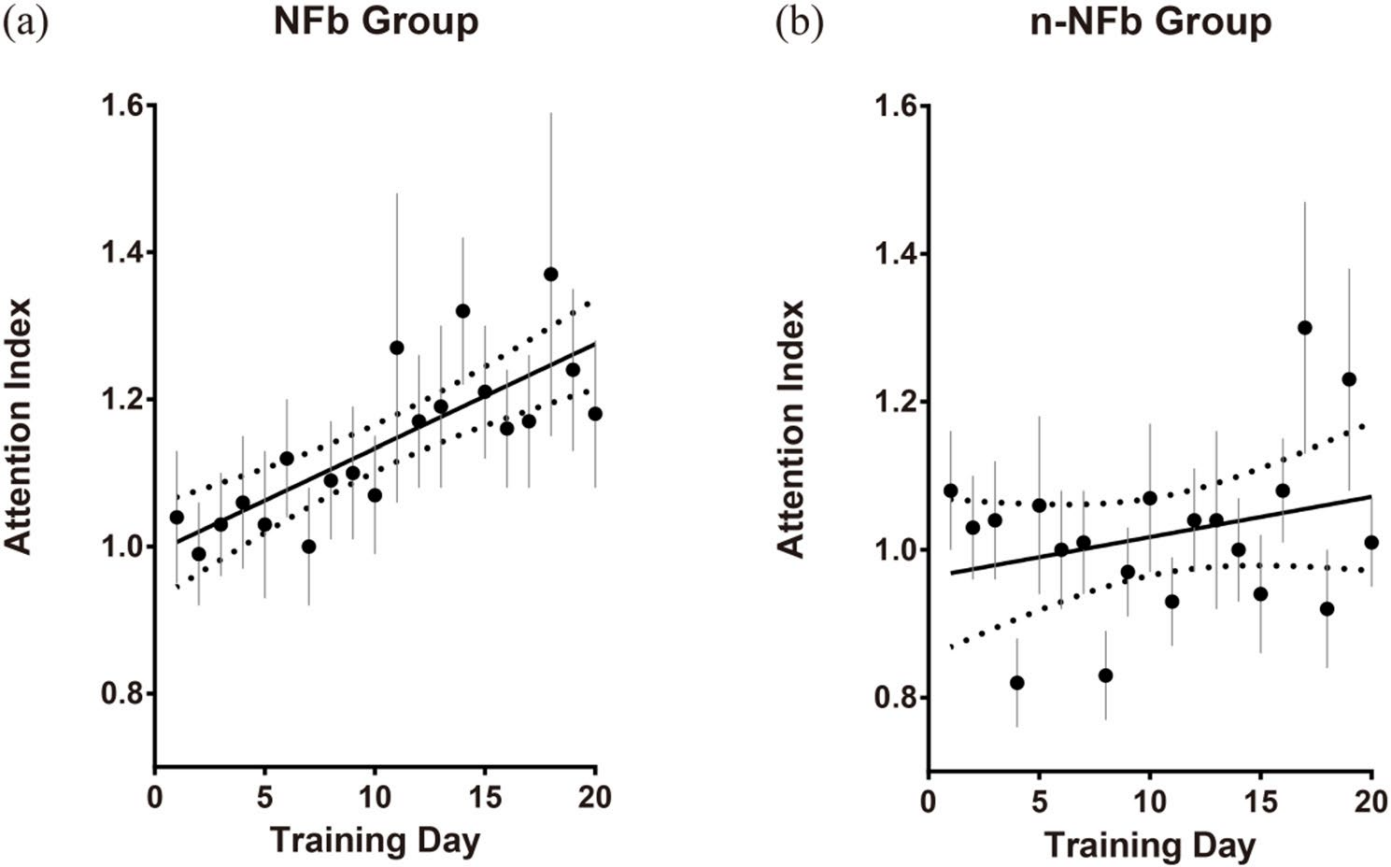
Linear regression results of the trends in Attention Index values relative to training days of both **a** NFb and **b** n-NFb group. Solid lines: linear regression trendlines; dashed lines: 95% Confidence Intervals; errorbars: standard error of the mean (SEM)

Daily averaged Attention Index values (see Chap. 2.4), indicating attentional level, were computed for both groups, with within-subject normalization applied using each participant’s daily averaged Attention Index baseline values. The Attention Index was derived from individualized beta range with lower bounds (11.44–13.0 Hz) and upper bounds (26.4–30.0 Hz), which fell within the conventional beta band while allowing meaningful individual variation. Linear regression was then conducted to examine the relationship between Attention Index and training days across groups (Fig. 3).

In the NFb group, a modest linear correlation that approached statistical significance was observed (*R²* = 0.619, *p* < 0.001), suggesting that neurofeedback training may contribute to improvements in attention as measured by the EEG Attention Index. In contrast, the n-NFb group showed no evidence of a linear relationship between Attention Index and training duration (*R²* = 0.082, *p* = 0.220). To further validate group differences in correlation strength, Fisher’s z-transformation was applied. The analysis revealed that the Pearson correlation coefficient in the NFb group (*r* = 0.780, *n* = 36) was significantly greater than that in the n-NFb group (*r* = 0.290, *n* = 34; *Z* = 2.983, *p* = 0.001). Taken together, these findings support the contributive role of neurofeedback in shaping attention-related dynamics during training.

## Discussion

This study investigated the efficacy of a video game-based digital therapeutic with EEG-informed real-time neurofeedback for improving attention in children with ADHD. While both intervention groups showed significant improvements in attentional engagement, children receiving real-time EEG-based feedback demonstrated advantages. Specifically, the NFb group showed greater gains in sustained attention tasks and outperformed controls both during training and on subsequent attentional assessments. These findings highlight the potential of integrating neural feedback into digital therapeutics to enhance individualized cognitive training for pediatric ADHD.

This study provides the first empirical evidence that digital therapeutic with portable real-time EEG neurofeedback can enhance attentional training in children with ADHD. Compared with standard training, children receiving neurofeedback showed greater gains in sustained attention tasks, superior performance in TOVA, and improvements in EEG-derived Attention Index. While attention improvements were observed both in TOVA and parental SNAP-IV ratings, only the objective measures showed significant group differences, likely reflecting their higher sensitivity compared with subjective parental reports. Furthermore, although previous studies have demonstrated the usefulness of neurofeedback [20, 29], they typically relied on complex, laboratory-grade EEG systems. Our study is the first to validate that a portable, single-channel device can also yield measurable cognitive benefits. These findings highlight the promise of combining neurofeedback with digital therapeutics to deliver individualized, scalable, and accessible ADHD interventions.

The observed advantages of neurofeedback are likely driven by the provision of real-time EEG rhythm feedback, which offers biologically grounded cues for self-regulation. Previous studies have highlighted the role of the prefrontal cortex in supporting executive functions such as inhibitory control and attentional switching (e.g., [3]). The EEG neurofeedback may have served as an external scaffold to reinforce these processes dynamically during gameplay [31]. Additionally, the application of individualized brain rhythms takes into account the substantial variability in EEG abnormalities observed in children with ADHD across temporal, spatial, and spectral domains [4]. This approach differs from most rhythm-based neurofeedback studies, which typically rely on fixed frequency ranges derived from healthy adult EEG data. Standardized parameters of this kind may reduce relevance for pediatric ADHD populations. Several recent studies employing fixed EEG bands have reported diminished or non-significant neurofeedback effects in ADHD, underscoring the need for more flexible and individualized methodological designs [7, 12, 18, 24]. Despite its promise, the use of individualized brain rhythms in neurofeedback research remains relatively limited. Further studies are needed to identify the most reliable signals and to optimize parameter selection in order to maximize the therapeutic potential of neurofeedback interventions for ADHD.

In the current study, neurofeedback appeared particularly effective in tasks requiring rapid stimulus evaluation and response inhibition, such as the egg-hitting task. By contrast, improvements in sustained attention tasks, such as the number-approach task, were relatively modest. As both tasks were designed to be equally weighted and carried no predefined priority, this asymmetry may reflect task-specific sensitivity to the neurofeedback signal. The egg-hitting task requires rapid stimulus evaluation and response inhibition—cognitive functions closely associated with fluctuations in frontal EEG activity and commonly targeted in neurofeedback paradigms. In contrast, the number-approach task relies more on sustained attention and visual–motor coordination, which may be less directly modulated by the neurofeedback mechanism employed in this study. This pattern of selective enhancement suggests that neurofeedback may preferentially strengthen specific components of attentional control, particularly those involving inhibitory regulation. This interpretation is further supported by the superior performance of the NFb group on the No-Go trials, which also depend on effective response inhibition.

From a clinical perspective, our results support the feasibility and utility of neuroadaptive digital therapeutics as a scalable, low-burden alternative or complement to traditional ADHD treatments. The use of single-channel EEG offers a cost-effective and user-friendly platform for real-world applications, particularly in resource-limited settings. Note that the individual beta rhythm in this study should be regarded as an operational signal for closed-loop neurofeedback training, while its potential as a validated neural biomarker of attention requires further studies.

This study has several limitations. First, participants were medication-free and with IQ ≥ 70 to minimize potential confounding effects and ensure task feasibility, which may limit the generalizability of the findings to children with more severe ADHD or to those receiving medication. Accordingly, the study provides a proof-of-concept demonstration of an EEG-informed neuroadaptive digital therapeutic while applicability in broader clinical population requires further studies. Second, Although the sex distribution in the current study (78% and 86% male) was broadly consistent with epidemiological data (~ 82%; [19]), the predominance of male participants and the small number of females limited our ability to examine sex-specific effects; accordingly, gender was considered only as a covariate in exploratory analyses and did not show a significant main effect. Third, the result centered on the neuroadaptive intervention as a whole, further studies were required to disentangle EEG-specific contributions from enhanced contingency management, as well as interpretation of neural mechanisms. Finally, the intervention period was relatively short (one month), and long-term effects remain unknown.

Future studies should extend intervention duration, incorporate multi-channel EEG for finer-grained neural monitoring, and evaluate the transfer of attentional improvements to everyday functioning in broader population. Moreover, adaptive algorithms informed by machine learning and larger datasets may allow for more precise tailoring of game difficulty to individual neurocognitive dynamics. Ultimately, the integration of neurophysiological monitoring with personalized feedback holds promise for developing next-generation digital therapeutics that are both effective and widely accessible.

## Conclusions

This study provides converging behavioral and physiological evidences that video game-based digital therapeutics with EEG-informed real-time neurofeedback can enhance attention in children with ADHD. Compared to standard gameplay, the neuroadaptive version led to greater improvements in attentional performance. These findings highlight the potential of portable neuroadaptive systems as accessible and personalized interventions for pediatric ADHD.

## Data Availability

The datasets used and/or analysed during the current study are available from the corresponding author on reasonable request.

## Abbreviations

NFb: Neurofeedback
n-NFb: Non-neurofeedback

## References

[1] American Psychiatric Association. Diagnostic and statistical manual of mental disorders: DSM-5. Arlington: American Psychiatric Association; 2013.

[2] Arns M. EEG-based personalized medicine in ADHD: individual alpha peak frequency as an endophenotype associated with nonresponse. J Neurother. 2012;16(2):123–41.

[3] Benchenane K, Tiesinga PH, Battaglia FP. Oscillations in the prefrontal cortex: a gateway to memory and attention. Curr Opin Neurobiol. 2011;21(3):475–85. 21429736 10.1016/j.conb.2011.01.004

[4] Chen H, Song Y, Li X. Use of deep learning to detect personalized spatial-frequency abnormalities in EEGs of children with ADHD. J Neural Eng. 2019;16(6):066046. 31398717 10.1088/1741-2552/ab3a0a

[5] Chiu HJ, Sun CK, Fan HY, Tzang RF, Wang MY, Cheng YC, et al. Surface electroencephalographic neurofeedback improves sustained attention in ADHD: a meta-analysis of randomized controlled trials. Child Adolesc Psychiatry Ment Health. 2022;16(1):104. 36536438 10.1186/s13034-022-00543-1 PMC9764556

[6] Cohen J. Statistical power analysis for the behavioral sciences. New York: Routledge; 2013.

[7] Cortese S, Ferrin M, Brandeis D, Holtmann M, Aggensteiner P, Daley D, et al. Neurofeedback for attention-deficit/hyperactivity disorder: meta-analysis of clinical and neuropsychological outcomes from randomized controlled trials. J Am Acad Child Adolesc Psychiatry. 2016;55(6):444–55. 27238063 10.1016/j.jaac.2016.03.007

[8] Enriquez-Geppert S, Smit D, Pimenta MG, Arns M. Neurofeedback as a treatment intervention in ADHD: current evidence and practice. Curr Psychiatry Rep. 2019;21(6):46. 31139966 10.1007/s11920-019-1021-4 PMC6538574

[9] Faul F, Erdfelder E, Buchner A, Lang AG. Statistical power analyses using G*Power 3.1: tests for correlation and regression analyses. Behav Res Methods. 2009;41(4):1149–60. 19897823 10.3758/BRM.41.4.1149

[10] Forbes GB. Clinical utility of the test of variables of attention (TOVA) in the diagnosis of attention-deficit/hyperactivity disorder. J Clin Psychol. 1998;54(4):461–76. 9623751 10.1002/(sici)1097-4679(199806)54:4<461::aid-jclp8>3.0.co;2-q

[11] Gau SSF, Shang CY, Liu SK, Lin CH, Swanson JM, Liu YC, et al. Psychometric properties of the Chinese version of the Swanson, Nolan, and Pelham, version IV scale–parent form. Int J Methods Psychiatr Res. 2008;17(1):35–44. 18286459 10.1002/mpr.237 PMC6878250

[12] Geladé K, Bink M, Janssen TW, van Mourik R, Maras A, Oosterlaan J. An RCT into the effects of neurofeedback on neurocognitive functioning compared to stimulant medication and physical activity in children with ADHD. Eur Child Adolesc Psychiatry. 2017;26(4):457–68. 27665293 10.1007/s00787-016-0902-x PMC5364239

[13] Hao Z, He C, Ziqian Y, Haotian L, Xiaoli L. Neurofeedback training for children with ADHD using individual beta rhythm. Cogn Neurodyn. 2022;16(6):1323–33. 36408061 10.1007/s11571-022-09798-y PMC9666577

[14] Huang S, Zhang T, Lu Q, Xiong X, Liu Z, Sun D. Clinical study on the intervention effect of digital therapy on children with attention deficit hyperactivity disorder (ADHD). Sci Rep. 2024;14(1):23733. 39390049 10.1038/s41598-024-73934-3 PMC11467183

[15] Klimesch W. EEG alpha and theta oscillations reflect cognitive and memory performance: a review and analysis. Brain Res Rev. 1999;29(2–3):169–95. 10209231 10.1016/s0165-0173(98)00056-3

[16] Kollins SH, DeLoss DJ, Cañadas E, Lutz J, Findling RL, Keefe RS, et al. A novel digital intervention for actively reducing severity of paediatric ADHD (STARS-ADHD): a randomised controlled trial. Lancet Digit Health. 2020;2(4):e168–78. 33334505 10.1016/S2589-7500(20)30017-0

[17] Liu NH, Chiang CY, Chu HC. Recognizing the degree of human attention using EEG signals from mobile sensors. Sens (Basel). 2013;13(8):10273–86. 10.3390/s130810273 PMC3812603 23939584

[18] Logemann HA, Lansbergen MM, Van Os TW, Böcker KB, Kenemans JL. The effectiveness of EEG-feedback on attention, impulsivity and EEG: a sham feedback controlled study. Neurosci Lett. 2010;479(1):49–53. 20478360 10.1016/j.neulet.2010.05.026

[19] Martin J, Langley K, Cooper M, Rouquette OY, John A, Sayal K, et al. Sex differences in attention-deficit hyperactivity disorder diagnosis and clinical care: a national study of population healthcare records in Wales. J Child Psychol Psychiatry. 2024;65(12):1648–58. 38864317 10.1111/jcpp.13987

[20] Micoulaud-Franchi JA, Geoffroy PA, Fond G, Lopez R, Bioulac S, Philip P. EEG neurofeedback treatments in children with ADHD: an updated meta-analysis of randomized controlled trials. Front Hum Neurosci. 2014;8:906. 25431555 10.3389/fnhum.2014.00906 PMC4230047

[21] Mikami K, Miyajima T, Nishino R, Kawazoe N, Kinoshita Y, Okada T et al. Efficacy and safety of SDT-001, a dual‐task digital device, in managing ADHD symptoms in children and adolescents: a phase 3 randomized, standard treatment–controlled study. Psychiatry Clin Neurosci. 2025. 10.1111/pcn.13833 PMC12319664 40317565

[22] Nazarova VA, Sokolov AV, Chubarev VN, Tarasov VV, Schiöth HB. Treatment of ADHD: drugs, psychological therapies, devices, complementary and alternative methods as well as the trends in clinical trials. Front Pharmacol. 2022;13:1066988. 36467081 10.3389/fphar.2022.1066988 PMC9713849

[23] Oh S, Choi J, Han DH, Kim E. Effects of game-based digital therapeutics on ADHD in children and adolescents as assessed by parents or teachers: a systematic review and meta-analysis. Eur Child Adolesc Psychiatry. 2024;33(2):481–93. 36862162 10.1007/s00787-023-02174-z

[24] Ogrim G, Kropotov J, Hestad K. The quantitative EEG theta/beta ratio in ADHD and normal controls: sensitivity, specificity, and behavioral correlates. Psychiatry Res. 2012;198(3):482–88. 22425468 10.1016/j.psychres.2011.12.041

[25] Peterson BS, Trampush J, Maglione M, Bolshakova M, Rozelle M, Miles J, et al. Treatments for ADHD in children and adolescents: a systematic review. Pediatrics. 2024;153(4):e2024065787. 38523592 10.1542/peds.2024-065787

[26] Raven J. Raven progressive matrices. In: McCallum RS, editor. Handbook of Nonverbal Assessment. Boston: Springer US; 2003. pp. 223–37.

[27] Salari N, Ghasemi H, Abdoli N, Rahmani A, Shiri MH, Hashemian AH, et al. The global prevalence of ADHD in children and adolescents: a systematic review and meta-analysis. Ital J Pediatr. 2023;49(1):48. 37081447 10.1186/s13052-023-01456-1 PMC10120242

[28] Serrano-Barroso A, Siugzdaite R, Guerrero-Cubero J, Molina-Cantero AJ, Gomez-Gonzalez IM, Lopez JC, et al. Detecting attention levels in ADHD children with a video game and a single-channel BCI headset. Sens (Basel). 2021;21(9):3221. 10.3390/s21093221 PMC8124980 34066492

[29] Sitaram R, Ros T, Stoeckel L, Haller S, Scharnowski F, Lewis-Peacock J, et al. Closed-loop brain training: the science of neurofeedback. Nat Rev Neurosci. 2017;18(2):86–100. 28003656 10.1038/nrn.2016.164

[30] Swanson JM, Kraemer HC, Hinshaw SP, Arnold LE, Conners CK, Abikoff HB, et al. Clinical relevance of the primary findings of the MTA: success rates based on severity of ADHD and ODD symptoms at end of treatment. J Am Acad Child Adolesc Psychiatry. 2001;40(2):168–79. 11211365 10.1097/00004583-200102000-00011

[31] Van Doren J, Arns M, Heinrich H, Vollebregt MA, Strehl U, Loo SK. Sustained effects of neurofeedback in ADHD: a systematic review and meta-analysis. Eur Child Adolesc Psychiatry. 2019;28(3):293–305. 29445867 10.1007/s00787-018-1121-4 PMC6404655

[32] Wolfers T, Beckmann CF, Hoogman M, Buitelaar JK, Franke B, Marquand AF. Individual differences vs the average patient: mapping heterogeneity in ADHD using normative models. Psychol Med. 2020;50(2):314–23. 30782224 10.1017/S0033291719000084 PMC7083555

[33] Zhao X, Page TF, Altszuler AR, Pelham WE, Kipp H, Gnagy EM, et al. Family burden of raising a child with ADHD. J Abnorm Child Psychol. 2019;47:1327–38. 30796648 10.1007/s10802-019-00518-5

